# HOOK ASSISTED REDUCTION IN CEPHALOMEDULLARY NAILING WITHOUT TRACTION TABLE

**DOI:** 10.1590/1413-785220243202e274533

**Published:** 2024-06-24

**Authors:** Cagatay Tekin, Burak Gunaydin, Mesut Karıksız

**Affiliations:** 1 Istanbul Cam and Sakura City Hospital, Orthopaedics and Traumatology Department, Turkey.; 2 Tekirdag Namik Kemal University Medical Faculty, Orthopaedics and Traumatology Department, Turkey.; 3 Başakşehir Cam and Sakura City Hospital, Orthopedic and Traumatology, Istanbul, Turkey.

**Keywords:** Femoral Fractures, Surgical Procedures, Operative, Surgical Hooks, Developing Countries, Fraturas do Fêmur, Procedimentos Cirúrgicos Operatórios, Ganchos Cirúrgicos, Países em Desenvolvimento

## Abstract

**Introduction:**

Proximal femoral nailing for intertrochanteric femur fracture is sometimes a challenging procedure without a traction table, especially if complicated fracture pattern. We aimed to overcome this difficulty with the hook.

**Materials and Methods:**

A retrospective study of 60 patients. 28 of the patients reduction was necessitated with a hook (group 1). The other patients did not need to use this technique (group 2, n=32). The collo-diaphyseal angle, lag screw placement, and tip-apex distance were measured using radiographs.

**Results:**

There were statistically significant differences between the two groups regarding the Garden Alignment Index, postoperative collo-diaphyseal angle measurements, and tip-apex distance. The Garden Alignment Index was found as 163.92 degrees (dg.) In the frontal plane in group 1, and 154.78 dg in group 2, respectively. In group 1, the tip-apex distance was 16.05 cm, whereas it was 25.32 cm in group 2. The collo-diaphyseal angle was 133.1º in group 1, and 128.65º in group 2.

**Conclusions:**

The hook-assisted reduction is beneficial when operating without a traction table; however, it can also be a part of the surgeons’ equipment even when operating on a traction table. When difficulties in obtaining an ideal anatomical reduction in displaced intertrochanteric femoral fractures, we suggest using the hook-assisted reduction technique. ***Level of Evidence III; Case-control Study.***

## RESUMO

**Introdução:**

Frequentemente, a fixação do fêmur proximal para fratura intertrocantérica do fêmur sem uma mesa de tração é um procedimento desafiador, especialmente se o padrão da fratura for complicado. O objetivo foi superar essa dificuldade utilizando um gancho.

**Materiais e métodos:**

Trata-se de um estudo retrospectivo de 60 pacientes. Em 28 desses, a redução foi necessária com um gancho (grupo 1). Os outros pacientes não precisaram usar essa técnica (grupo 2, n=32). O ângulo colo-diafisário, a colocação do parafuso lag e a distância ponta-ápice foram medidos por meio de radiografias.

**Resultados:**

Houve diferenças estatisticamente significativas entre os dois grupos com relação ao Índice de Alinhamento de Garden, às medidas do ângulo colo-diafisário pós--operatório e à distância ponta-ápice. O índice de alinhamento de Garden foi de 163,92 graus (dg.) No plano frontal no grupo 1 e 154,78 dg no grupo 2, respectivamente. No grupo 1, a distância ponta-ápice foi de 16,05 cm, enquanto no grupo 2 foi de 25,32 cm. O ângulo colo-diafisário foi de 133,1 graus no grupo 1 e 128,65 graus no grupo 2.

**Conclusão:**

A redução assistida por gancho é benéfica quando se opera sem uma mesa de tração; no entanto, ela também pode fazer parte do equipamento do cirurgião mesmo quando se opera em uma mesa de tração. Quando houver dificuldades em obter uma redução anatômica ideal em fraturas femorais intertrocantéricas deslocadas, sugerimos o uso da técnica de redução assistida por gancho. ***Nível de Evidência III; Estudo de Caso-controle.***

## INTRODUCTION

Closed anatomical reduction of displaced intertrochanteric femur fractures (IFFs) has challenged orthopedic surgeons due to the broken medial hinge. The displacement of broken fragments by strong muscles may not allow the fracture to be reduced.^1,2^ A fracture table is a part of the technique in most countries; however, limited centers in developing countries own a particular table. Limited surgical hints are available for intertrochanteric fractures.^3,4^

Some auxiliary techniques for reducing unstable IFFs such as Steinmann pins,^3^ various types of bone clamps, and even some authors advised for open reduction after unsuccessful attempts. Additional surgeries can be anticipated if the reduction is not appropriately made.^5^

In this study, we aimed to evaluate the hook-assisted reduction technique, which we have used since 2015 in patients with IFFs if the reduction was difficult.

## PATIENTS AND METHODS

### Patients selection

The Local Ethics Committee approved this study (Date: 24/09/2019, 2019/156/09/16).

Between December 2015 and March 2019, 66 patients with IFFs who underwent osteosynthesis with cephalomedullary nailing were identified. One patient died in the early postoperative period, and five did not attend outpatient follow-ups after discharge. Accordingly, 60 patients were included in the study and evaluated retrospectively. All patients provided written informed consent to participate. Fractures were classified according to the A.O. Foundation/Orthopaedic Trauma Association System. The inclusion criteria were as follows: patients over 18 years, treatment with cephalomedullary nail in the lateral decubitus position without a traction table, and at least six months of out-patient follow-up. Patients were excluded if they had A1.1 type proximal femur fractures, but 1.2 and 1.3 were included because of displacement of these type fractures whether they're stable or not collo-diaphyseal angle distortion less than 5 degrees from the opposite side, patients with pathologic fractures, patients with follow-up less than six months.

## METHODS

**Figure 1:**
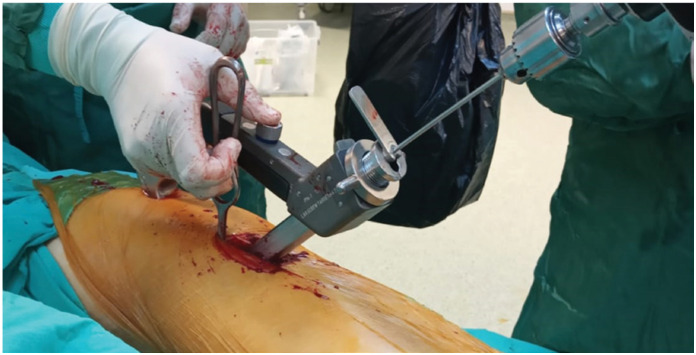
Application of the hook in hook-assisted reduction methods.

**Figure 2:**
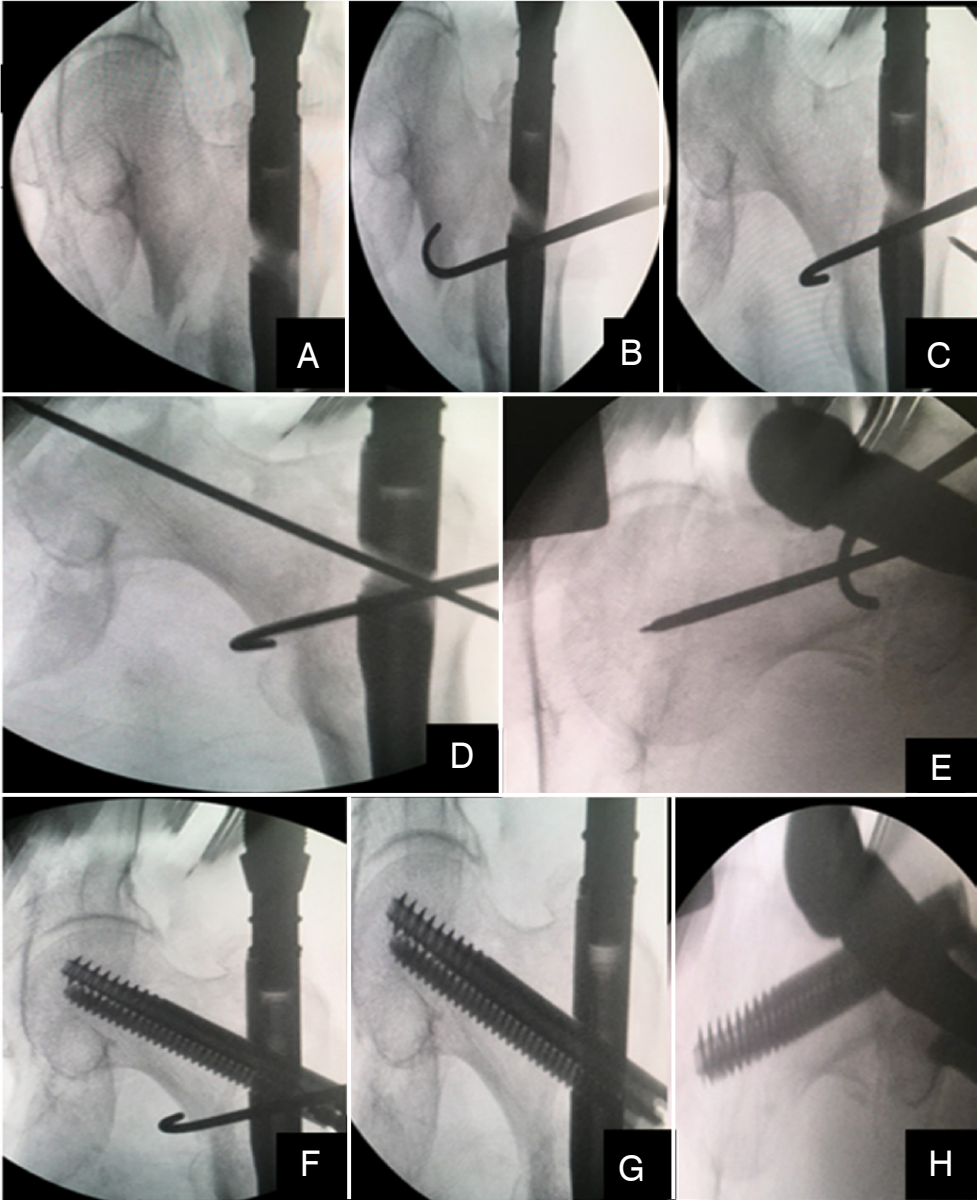
Fluoroscopic images: (A) Anteroposterior (AP) view showing fracture displacement prior to reduction; (B) AP view before hook-assisted reduction (C) AP view after hook-assisted reduction (D) AP view after proximal femoral nail guide wire was applied (E) Lateral (frog-leg) image after proximal femoral nail guide wire was applied (F) AP image when a two-screw cephalomedullary nail was applied (G) AP fluoroscopy image of two-screw cephalomedullary nail after removing the hook (H) Frog-leg image of two-screw cephalomedullary nail.

**Figure 3:**
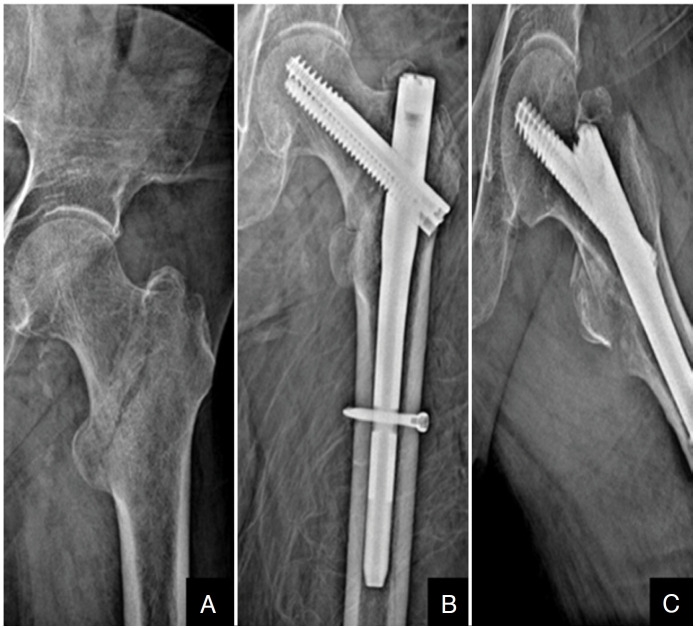
(A) 92-year-old patient in the hook-assisted reduction group, preoperative AP fracture radiograph in traction; (B) AP postoperative radiography of a patient with a two-screw cephalomedullary nail; (C) Postoperative frog-leg radiography of a patient with a two-screw cephalomedullary nail.

The Integrated Compression Screw cephalomedullary nail (interTAN, Smith & Nephew, Memphis, TN) was used for internal fixation. All operations were performed in the lateral decubitus position without a traction table. If the reduction is appropriate, the nail is inserted. The hook-assisted reduction technique was initiated if the alignment was not acceptable despite three consecutive attempts. While one orthopedic surgeon used this technique, a control group was formed with the permission of the other surgeon, who did not use hook-assisted reduction but attempted any other auxiliary tools. He was able to use the hook through existing incisions. If the hook could not be utilized using previous incisions, an additional 2 cm incision was done laterally to provide access to the fracture (Figure 1). A case example showing hook-assisted reduction and surgical fluoroscopy images during the reduction and application of the nail can be found in Figure 2a-g. Preoperative traction radiography, postoperative A.P., and lateral radiographs of the patient can be found in Figure 3a-c.

### Study Protocol

Age, sex, fractured side, follow-up, and fracture type were determined. Mobilization, weight-bearing, and union data of the patients were recorded. The collo-diaphyseal angle, Garden Alignment Index (frontal), tip-apex distance, the quadrant of the helical blade according to Cleveland and Bosworth,^6^ and Ikuta's reduction subgroups were determined.^7^ The Herman criteria were used for the quality of reduction.^8^

Accordingly, for the reduction to be considered appropriate, it was assumed that there was no varus position, and displacement between the medial cortices measured on A.P. and lateral radiographs should be less than 5 mm or near at sight. If two of these conditions were met, the reduction was assessed as "good," if one was completed, as "acceptable," if no criteria were met, as "poor." The union was determined by a single surgeon with radiographs taken in the follow-up of patients. Sectra UniView (Sweden, version 20.2.14.3442) was used in the measurements. The presence of union was defined as the presence of callus formation as a result of bridging at least three cortices on A.P. and lateral radiographs. Complications and mortality were recorded in outpatient clinic follow-up.

### Statistical Analysis

Statistical analyses were performed using the Statistical Package for the Social Sciences (SPSS, Chicago, Illinois, USA), version 23.0 software. A standard distribution test was performed on all data. For the comparison of quantitative data, Student's t-test was used for those with normal distribution, and the Mann-Whitney U test was used for non-parametric data. Fisher's Chi-square test was used to compare qualitative data. Statistical significance was set as p < 0.05.

## RESULTS

The patients were divided into two groups: those who underwent the hook method (group 1, n=28) and those without the hook method (group 2, n=32). Average values of age, follow-up time, sex, and side (Table 1) were summarized.

**Table 1:** Average values of age, follow-up time, sex, side distribution by groups.

		Group 1 n=28	Group 2 n=32	^p^
Age (Year) mean ± SD, (Min-Max)		72.25 ± 18.91 (27-92)	77.03 ± 14.14 (32-95)	0.553
Follow-up time (month) mean ± SD, (Min-Max)		16.53 ±11.60 (6-45)	16.65 ± 11.35 (6-40)	0.97
Sex (%)	Female	16 (57.1%)	13 (40.6%)	0.3
	Male	12 (42.9%)	19 (59.4 %)	
Side (%)	Left	16 (57.1%)	18 (56.3%)	0.576
	Right	12 (42.9%)	14 (43.8%)	

Fractures were classified according to type 31. Groups in Table 2 summarize A.O. Fracture types. Patients with 31AO-A1-1 fractures were excluded from the study.

**Table 2:** OTA / AO fracture classification by groups.

	Group 1 n=28 (%)	Group 2 n=32 (%)	p
31AO-A1-2	8 (28.6%)	8 (25%)	0.91
31AO-A1-3	5 (17.9%)	3 (9.4%)	
31AO-A2-1	3 (10.7%)	2 (6.3%)	
31AO-A2-2	4 (14.3%)	6 (18.8%)	
31AO-A2-3	1 (3.6%)	2 (6.3%)	
31AO-A3-1	3 (10.7%)	4 (12.5%)	
31AO-A3-2	0 (0%)	1 (3.1%)	
31AO-A3-3	4 (14.3%)	6 (18.8%)	

The Garden Alignment Index in the frontal plane was 163.92 degrees in group 1, and 154.78 degrees in group 2 (p<0.001). The tip-apex distance was determined as 16.05 mm in group 1 and 25.32 mm in group 2 (p=0.001). The mean collo-diaphyseal angle was 133.1 degrees in group 1 and 128.65 degrees in group 2 (p=0.032) (Table 3).

**Table 3:** The mean collo-diaphyseal angle, Garden Alignment Index (frontal plane) measurements, and tip-apex distance measurements according to the groups.

	Group 1, mean ± SD, (Min-Max)	Group 2, mean ± SD, (Min-Max)	p
Collo-diaphyseal angle (degrees)	133.1 ± 6.96 (116-145)	128.65 ± 7.36 (103-138)	0.032
Garden Alignment Index frontal (degrees)	163.92 ± 5.49 (147-171)	154.78 ± 6.35 (135-165)	<0.001
Tip-apex distance (mm)	16.05 ± 7.23 (3-25)	25.32 ± 12.23 (2-62)	0.001

**Figure 4:**
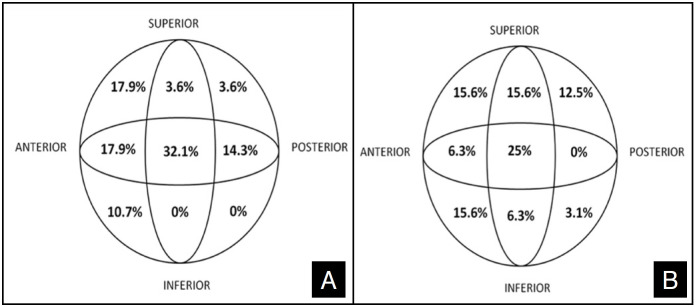
The positions of the helical blade on the quadrant of the helical blade according to Cleveland and Bosworth (A) group 1 (hook method) (B) group 2 (no hook).

The quadrant of the helical blade, which was advanced to the femoral neck, is shown in Figures 4 a-b. The percentage of patients in the recommended quadrants in the postoperative radiographs was 32.1% for group 1 and 31.3% for group 2. The implant was in the superior-posterior quadrant in 3.6% of patients in group 1, the quadrant in which the implant should not be placed, whereas this ratio was 12.5% i n group 2. Patients who underwent arthroplasty with cut-out complications were those whose implants were in the superior-posterior and central-posterior quadrants in group 1. However, it was in the superior-posterior quadrant in all patients in group 2.

According to Herman's criteria, we accepted 130 degrees as a cut-off value for varus alignment; a good reduction was seen in 20 patients, and an acceptable reduction was seen in six patients (varus alignment in four patients, fracture interval over 5 mm in two patients) in group 1. A good reduction was observed in 20 patients, and an acceptable reduction was observed in 10 patients (varus alignment in six patients, fracture interval over 5 mm in four patients) in group 2. The poor reduction was detected in two patients in both groups (Table 4).

**Table 4:** Distribution of groups according to the Herman criteria and Ikuta classification.

			Group 1 n=28 (%)	Group 2 n=32 (%)	p
Herman Criteria	Good Reduction		20 (71.4%)	20 (62.5%)	0.849
	Acceptable	Varus alignment	4 (14.3%)	6 (18.8%)	
		Fracture range	2 (7.1%)	4 (12.5%)	
	Poor reduction		2 (7.1%)	2 (6.3%)	
Ikuta Classification	Normal		12 (42.9%)	14 (43.8%)	0.619
	Posterior		6 (21.4%)	4 (12.5%)	
	Anterior		12 (34.7%)	14 (43.8%)	

According to the Ikuta classification, 12 patients were typical subtypes, six were posterior, and 12 had anterior subtypes in group 1. In group 2, 14 patients were typical subtypes, four were posterior subtypes, and 14 were anterior subtypes (Table 4). All those with cut-out complications were classified in the posterior subtype according to the Ikuta classification.

Mean mobilization, weight-bearing, and fracture union times were summarized in Table 5. General complications and mortality distribution of the Groups can be found in Table 6. No deep infections or vascular and nerve lesions were detected in any patients.

**Table 5:** Mean mobilization, weight-bearing, and fracture union times by groups.

	Group 1 mean ± SD, (Min-Max)	Group 2 mean ± SD, (Min-Max)	p
Mobilization	1.82 ± 0.81 (1-4)	1.96 ± 0.78 (1-4)	0.425
Weight bearing	3.46 ± 1.52 (2-6)	3.96 ± 1.44 (2-6)	0.180
Union	7.03 ± 2.48 (4-12)	7.31 ± 2.46 (4-12)	0.503

**Table 6:** General complications and mortality distribution by groups.

Complications	Group 1 n=28 (%)	Group 2 n=32 (%)	p
Mortality	2 (7.1%)	2 (6.3%)	0.89
DVT	2 (7.1%)	2 (6.3%)	0.89
Cut-out	2 (7.1%)	3 (9.4%)	0.755
Varus collaps*	1 (3.6%)	1 (3.1%)	0.923
Re-operation	3 (10.7%)	4 (12.5%)	0.830
Superficial infection	1 (3.6%)	0 (0%)	0.467

* Excluding patients with cut-out.

A statistically significant difference was found in the Garden Alignment Index, tip-apex distance, and collo-diaphyseal angle measurements.

## DISCUSSION

Displaced IFFs are not uncommon fractures and several methods were described.^9^ The critical point for the successful treatment of IFF, so hip fractures in the elderly, is to obtain stable geometry and rigid internal fixation for treatment and encourage patients to mobilize as early as possible.

IFFs are the most common type of proximal femoral fractures and can face various stress rates due to body weight and muscles around the hip. In this region, the reduction can occasionally be difficult due to the push-pull forces caused by the muscles. For the same reason, some surgeons experience reduction problems. The hook-assisted reduction is used, especially in cases where reduction is challenging. Internal fixation is the preferred surgical treatment for IFFs.^10^ However, performing and maintaining a proper alignment before placing the implant can be difficult for displaced IFFs. Techniques have been described to prevent this complication.^11^ Chun et al. described a method in which they reduced with one or two Steinmann pins used in the sagittally unstable IFFs.^3^ In this study, hook-assisted reduction was used in cases where reduction could not be achieved with traction and rotation maneuvers.

Short intramedullary nails can be applied with or without a fracture table. Although most surgeons prefer to use a traction table, there are instances where it is not available. Availability problems make surgeons find alternative ways, especially in developing countries.^12^ In 2016, Sahin et al. compared femoral nailing procedures in unstable IFFs using a traction table or manual traction. As a result, they determined that despite the increase in the number of surgical assistants required for manual traction, the preparation and the total anesthesia times were shorter using manual traction.^13^

In the surgical treatment of IFFs, the appropriate reduction must be achieved before starting nailing.^14^ In some cases, although all means of reduction are being used, such as increased traction and the addition of rotational maneuvers, a sufficient reduction cannot be achieved. We used the "hook-assisted method" in these cases to provide an acceptable reduction.

Ikuta classification was used in the postoperative lateral radiographs to evaluate the head-neck segment's alignment according to the distal fracture fragment. It is divided into standard (central), posterior, and anterior subtypes.^3^ It was in a normal position in 42.9% in group 1 and 43.8% of patients in group 2. The cut-out complication was seen in patients with Ikuta posterior subtype.

In group 1, the cut-out rate was 7.1% (n=2), whereas in group 2, it was 9.4% (n=3). The literature shows that the cut-out ratio of intramedullary implants is 8%.^15^ Our series observed an 8.3% overall complication rate when all patients were included.

The study has some limitations, such as being a retrospective study. No functional score has been added, and the last one limited number of patients and short follow-up can also be counted.

In displaced intertrochanteric femoral fractures, difficulties in obtaining an ideal anatomical reduction that sometimes may lead to malreduction have been challenging for orthopedic surgeons. This challenge can get more complicated when assisting apparatus such as a traction table is unavailable, which may be the case in developing countries. The hook-assisted reduction is beneficial when operating without a traction table^16^; however, it can also be a part of the surgeons’ equipment even when operating on a traction table.
